# Optimization of the Heterologous Expression of the Cannabinoid Type-1 (CB_1_) Receptor

**DOI:** 10.3389/fendo.2021.740913

**Published:** 2021-10-20

**Authors:** Viktória B. Horváth, Eszter Soltész-Katona, Éva Wisniewski, Anikó Rajki, Eszter Halász, Balázs Enyedi, László Hunyady, András Dávid Tóth, Gergő Szanda

**Affiliations:** ^1^ Department of Physiology, Semmelweis University, Budapest, Hungary; ^2^ MTA-SE Laboratory of Molecular Physiology, Eötvös Loránd Research Network, Budapest, Hungary; ^3^ MTA-SE Lendület Tissue Damage Research Group, Hungarian Academy of Sciences and Semmelweis University, Budapest, Hungary; ^4^ HCEMM-SE Inflammatory Signaling Research Group, Department of Physiology, Semmelweis University, Budapest, Hungary; ^5^ Department of Internal Medicine and Haematology, Semmelweis University, Budapest, Hungary

**Keywords:** CB_1_ receptor, receptor degradation, cannabinoids, weak promoters, heterologous expression, non-canonical signaling

## Abstract

The G protein-coupled type 1 cannabinoid receptor (CB_1_R) mediates virtually all classic cannabinoid effects, and both its agonists and antagonists hold major therapeutic potential. Heterologous expression of receptors is vital for pharmacological research, however, overexpression of these proteins may fundamentally alter their localization pattern, change the signalling partner preference and may also spark artificial clustering. Additionally, recombinant CB_1_Rs are prone to intense proteasomal degradation, which may necessitate substantial modifications, such as N-terminal truncation or signal sequence insertion, for acceptable cell surface expression. We report here that tuning down the expression intensity of the full-length CB_1_R reduces proteasomal degradation and offers receptor levels that are comparable to those of endogenous CB_1_ receptors. As opposed to high-efficiency expression with conventional promoters, weak promoter-driven CB_1_R expression provides ERK 1/2 and p38 MAPK signalling that closely resemble the activity of endogenous CB_1_Rs. Moreover, weakly expressed CB_1_R variants exhibit plasma membrane localization, preserve canonical G_i_-signalling but prevent CB_1_R-G_s_ coupling observed with high-expression variants. Based on these findings, we propose that lowering the expression level of G protein-coupled receptors should always be considered in heterologous expression systems in order to reduce the pressure on the proteasomal machinery and to avoid potential signalling artefacts.

## Introduction

The cannabinoid type-1 receptor (CB_1_R) is a G protein-coupled receptor (GPCR) that conveys both the therapeutic and the side-effects and of plant-derived phyto- and synthetic cannabinoids, and also mediates the actions of the body’s own cannabinoids (the endocannabinoids). CB_1_Rs play important regulatory role in virtually all central nervous areas (1, 2), whereas peripheral receptors are now well-documented contributors to the development of diet-induced obesity (3, 4), pancreatic β-cell dysfunction (5, 6), leptin and insulin resistance (7, 8) and to the complications of metabolic syndrome (9, 10). By virtue of such multifaceted actions under physiological and pathological conditions, both CB_1_R agonists and antagonists hold considerable therapeutic promise (11, 12) with encouraging new-generation ligands being under development (4, 13–15).

The heterologous expression of recombinant receptors is often the first step of drug development. Vector-driven expression in cell lines provides invaluable knowledge of receptor function and ligand characteristics. However, caution should be taken when interpreting *in vitro* data as high expression level of recombinant receptors may lead to altered localization, artificial dimerization or clustering of receptors (16–18) and may even cause membrane deformations (19). Moreover, high receptor amounts may alter the receptor-G protein stoichiometry and, in turn, bring about non-canonical signalling events, as reported in the case of A_1_ adenosine (20), α_2_-adrenergic (21) and, prominently, CB_1_ receptors (15).

Another possible drawback of receptor overexpression may be the intense degradation of the recombinant receptor *via* proteolytic pathways that are not significantly involved in receptor turnover normally. This certainly applies for the CB_1_R, in which the combination of an unusually long N-terminal tail and the lack of a signal sequence impedes normal co-translational translocation across the ER membrane and marks the recombinant receptor for proteasomal degradation (22, 23). This undesired degradation can be overcome by N-terminal truncation or by N-terminal insertion of a signal sequence, nevertheless, such manoeuvres may alter receptor maturation and trafficking in an unpredictable manner (23, 24).

In the light of the abovementioned pitfalls, heterologous expression of the CB_1_R and of other GPCRs ought to be optimized on a regular basis so as to minimize the risk of signalling artefacts and the overload of the proteasomal machinery. We speculated that simply lowering the expression level of the CB_1_R to close-to-endogenous amounts may provide such benefits. We report here that, in fact, reducing the transcription level of the recombinant CB_1_R is sufficient to reduce proteasomal degradation *and* to eliminate non-canonical signalling events without the need to modulate the receptor sequence itself.

## Materials and Methods

### Materials and Pharmacons

Unless otherwise noted, drugs were dissolved in sterile dimethyl sulfoxide (DMSO) (To avoid water absorption into the solvent, the sterile filtered DMSO was stored in small aliquots at -20°C.) The stock solutions were split into small aliquots and a maximum of 2 freeze-thaw cycles were allowed. In all experiments, the final concentration of DMSO was limited to 0.15%. (For the origin of chemicals, final concentrations and for further methodological details see **Supplementary Table I**).

### Cell Culture, Transfection and Cell Stimulation

GT1-7 cells (25) were from Sigma Aldrich, Neuro 2a and HEK 293 cells were from American Type Culture Collection (ATCC). All cells were cultured in Dulbecco’s Modified Eagle Medium (DMEM) containing 10% foetal bovine serum and supplemented with 100 μg/ml streptomycin and 100 U/ml penicillin (“complete DMEM”). (1.5 g/L NaHCO_3_ formulations were used exclusively to achieve pH 7.4 at 5% CO_2_ and 37°C.) Cells were plated into poly-L-lysine-coated 6-well or 12-well plates at a density of 6-7.5-10×10^4^/cm^2^ (GT1-7), 3-4×10^4^/cm^2^ (Neuro 2a) or 2-3×10^4^/cm^2^ (HEK 293) on day 1. GT1-7 cells were electroporated (1350 V, 30 ms; 1x) on the day of plating with the Neon Transfection system (Invitrogen) following the manufacturer’s protocol or transfected in Ultra-MEM with Lipofectamine 2000 (0.3 µL/cm^2^) for 6 h on day 2 and subsequently on day 3 with Lipofectamine LTX + Plus Reagent (0.5 µL/cm^2^ Lipofectamine LTX + 0.67 µL/µg total DNA Plus Reagent) in complete DMEM. HEK 293 cells were transfected on day 2 in Ultra-MEM with Lipofectamine 2000 (0.2-0.3 µL/cm^2^) or in complete DMEM with Lipofectamine LTX + Plus Reagent (0.6 µL/cm^2^ Lipofectamine LTX + 0.5 µL/µg total DNA Plus Reagent). Neuro 2a cells were transfected in complete DMEM using Lipofectamine LTX + Plus Reagent (0.6 µL/cm^2^ Lipofectamine LTX + 0.5 µL/µg total DNA Plus Reagent). Transfection of siRNA into Neuro 2a cells was performed as follows: on day 2 cells were treated with siRNA in Ultra-MEM for 6 h in the presence of Lipofectamine RNAiMAX (0.5 µL/cm^2^). Then, on day 3, cells were treated again with siRNA in complete DMEM for 6 h with Lipofectamine RNAiMax. Unless otherwise indicated, siRNA concentration was 20 nM and construct DNA amount was 0.05-0.07 µg/cm^2^/construct. Serum deprivation was performed by changing the complete medium to empty DMEM 14 h (GT1-7) or 4 h (Neuro 2a and HEK293) prior to experimentation. One hour prior to stimulation, medium was changed to DMEM + HEPES (Pan-Biotech) and cell stimulation was carried out in this incubation medium. In most of the experiments, 6-well plates and 12-well plates were snap frozen with liquid nitrogen and stored at -80°C until analysis. Passages numbers 4–30 were used. At all steps, bicarbonate-containing media were equilibrated at 37°C and 5% CO_2_ for at least 6 h before application.

### Constructs, siRNA

Human wild-type CB_1_R and Δ64 CB_1_R [lacking the N-terminal 1-64 amino acids (22)] were expressed in pcDNA3.1 vectors (driven by the early-immediate CMV promoter for high-level expression; sequence of the CMV promoter: *CGCCCCATTGACGCAAATGGGCGGTAGGCGTGTACGGTGGGAGGTCTATATAAGCAGAGCTGGTTTAGTGAACCGTCAGATC*. For mild heterologous expression a pEYFP-N1 backbone was modified by omitting YFP and changing the promoter to the human herpes simplex virus thymidine kinase (TK) promoter (in order to gain moderate expression of the transgene). Sequence of the thymidine kinase promoter was: *ATGACACAAACCCCGCCCAGCGTCTTGTCATTGGCGAATTCGAACACGCAGATGCAGTCGGGGCGGCGCGGTCCCAGGTCCACTTCGCATATTAAGGTGACGCGTGTGGCCTCGAACACCGAGCGACCCTGCAGCGACCCGCTTAA*. The cloning was performed with the help of the following enzymes: HindIII, AgeI, BamHI (Thermo Fisher Scientific). For fluorescent labelling, human wild-type and Δ64 CB_1_Rs were inserted into pEGFP-N1, or promoter modified (CMV to TK) pEYFP-N1 or promoter modified pEGFP expression vectors. Control (non-silencing) dsRNA sequences were designed based on the “C9-11” method (26). For dsRNA sequences, please refer to **Supplementary Table 2**.

### Western Blotting

The cultured cells were suspended in 4°C complete lysis buffer (*v.i.*) in a volume of 150-200 µL/9.5 cm^2^ growth area. After a 30 min incubation period on ice, insoluble material was removed by centrifugation at ~20.000 g (4°C for 10 min) and protein concentration was determined with BCA Assay (Thermo Fisher Scientific). The complete lysis buffer was based on a modified RIPA buffer containing: 150 mM NaCl, 1% sodium deoxycholate, 1% Triton X-100, 0.1% SDS, 1 mM EGTA 1 mM EDTA and 20 mM Tris-HCl (pH 7.35 at 4°C). This modified RIPA buffer was supplemented with 1 mM sodium orthovanadate, 1 mM phenylmethylsulfonyl fluoride, aprotinin, protease inhibitor cocktail, phosphatase inhibitor cocktail 1 and 2 (all 1:100). After the addition of 4x Laemmli sample buffer (Bio-Rad), proteins were separated in reducing mini or midi format Tris-glycine polyacrylamide gradient gels (4-15%, Bio-Rad). (Final concentration of β-mercaptoethanol in the samples was set to 5%). Alternatively, 2x Laemmli sample buffer with 10% of β-mercaptoethanol was used to suspend the cultured cells, so that the samples were directly loaded to the Tris-glycine polyacrylamide gradient gels. Proteins were then blotted onto nitrocellulose membranes using the Transblot-cell semi-dry transfer system with the fitting transfer packs (Bio-Rad).

Membranes were blocked with Tris buffered saline + 0.1% Tween-20 (TBST) supplemented with 5% milk and incubated overnight at 4°C with primary antibodies in TBST + 5% BSA + 0.1% sodium azide (for the list of antibodies, please see **Supplementary Table 3**). Horseradish peroxidase conjugated secondary antibodies (PerkinElmer) were diluted in TBST + 5% milk and incubated at room temperature for 1 h. Luminescence was measured with Azure 600 (Azure Biosystems, Dublin, CA, USA) chemiluminescence imaging device. West Pico Plus (ThermoFisher) or custom-made solution (100 mM Tris-Cl, 0.2 mM p-coumaric acid, 1.1 mM luminol, 2.6 mM H_2_O_2_, pH 8.5) was used as substrate for the peroxidase.

For densitometry, captured images were background subtracted with the apt module of Image J (NIH). The integrated density of individual protein bands was measured also with the Image J software and the ratio of phosphorylated to total protein (p/t ratio) was regarded as the degree of activation. Representative images were brightness and contrast adjusted with the appropriate modules of Image J (NIH) and blots were aligned in Adobe Illustrator. (Since chemiluminescent images have 16-bit depth, when needed, both high and low contrast versions of the same raw image are presented to provide a better representation. Please also note that western blots images comparing phosphorylated and total protein amounts of ERK1/2 and p38 MAPKs were exposed to identical conditions throughout the entire immunoblot process including image capture and brightness/contract adjustments. Therefore, phospho- and total blot images of different CB_1_R constructs within the same cell type are directly comparable.) Unless otherwise specified, data were normalized to the vehicle treated or the lowest agonist concentration treated TK-CB_1_R group.

### Protease Inhibition and ER-Stress Measurements

For the assessment of CB_1_R degradation, cells were treated with various protease and proteasome inhibitors or vehicle for 8 h in serum-free DMEM on day 4. For further details on pharmacons, please see **Supplementary Table 1**. To evaluate the intact-to-cleaved receptor relationship, the ratio of integrated densities between 50 to 100 kDa to that below 40 kDa was calculated.

### Measurement of Cytosolic [cAMP]

For BRET measurements, Neuro 2a or HEK 293 cells were transfected in suspension using Lipofectamine 2000 (Invitrogen; 0.5 μl/well) and plated on white poly-L-lysine coated 96-well plates in 50.000 cells/well density. The DNA amounts were 0.175 μg Epac-BRET sensor/well (27, 28) and 0.25 μg CB_1_ receptor construct/well. Before the BRET measurement, cells were serum starved for 3 hours. Experiments were performed on adherent cells 24 hours after the transfection using a Varioskan Flash multimode plate reader (Thermo Scientific, Waltham, MA). Prior to stimulation, the medium was changed to a modified Krebs-Ringer buffer containing 120 mM NaCl, 4.7 mM KCl, 1.2 mM CaCl_2_, 0.7 mM MgSO4, 10 mM glucose, and Na-HEPES 10 mM, pH 7.4, experiments were carried out at 37°C. The BRET measurements were started by adding cell-permeable coelenterazine *h* (Regis Technologies, Morton Grove, IL) to the wells at a final concentration of 5 μM. The luminescence intensities were recorded at 530 nm and 480 nm using filters (0.5 s/well). Since within the Epac-BRET sensor the intramolecular BRET *decreases* with the increase of [cAMP] (27), the 485 to 530 nm emission intensity ratio (BRET ratio) was regarded as a measure of [cAMP] (as opposed to the conventional 530:480 nm ratio). BRET ratios were normalized to the average of baseline (*i.e.* prior to the addition of any drug or vehicle).

### Confocal Microscopy

Cells expressing various GFP-tagged CB_1_R constructs were imaged on a spinning disk confocal imaging setup at room temperature (~26°C) using a Nikon Eclipse Ti2 microscope equipped with a CFI SR HP Plan Apochromat Lambda S 100XC silicon immersion objective lens, a Yokogawa CSU-W1 Spinning Disk unit, a Photometrics Prime BSI sCMOS camera and an Omicron LightHUB+ diode laser light engine. EGFP fluorescence was excited using the 488 laser line and emission was collected using a 525/20 bandpass filter (Chroma). Up to 70 Z-stack slices with a resolution of 0.3 µm were acquired per field of view with the NIS-Elements software (Nikon). Plasma membrane and cytosolic CB_1_R fluorescence in non-processed raw images were determined at approx. the bottom 1/3 in the z-axis (*i.e.* closer to the cell-coverslip interface) along a profile running through the cell. The first peak above the cell-free background was interpreted as plasmalemmal fluorescence whereas cytosolic florescence was defined as the intensity ‘below’ the plasma membrane, at a position exactly 1 µm towards the cell centre. For the purpose of demonstration, background subtraction and brightness/contrast adjustments were performed with Image J (NIH) on representative images.

### Data Analysis and Statistics

Means + s.e.m. or ± s.e.m. are shown, unless indicated otherwise. Data were obtained from at least 3 independent experiments or specified otherwise. In some experiments, minimal and maximal values have been uniformly excluded in all groups based on the ROUT method. For calculating significance of differences, one and two-way parametric or non-parametric ANOVA and post-hoc tests were applied, as appropriate. Concentration-response curves were fitted using the 3-parameter log[agonist] – response equation [(Y=Bottom + (Top-Bottom)/(1 + 10^(LogEC50-X)^)]. Data were analysed with Microsoft Excel (Microsoft), Image J (NIH) and GraphPad Prism 5 (GraphPad Software Inc.) software.

## Results

### Weak Promoter-Driven Expression of the Full-Length CB_1_R Provides Close-to-Endogenous Receptor Levels

**Figure 1:**
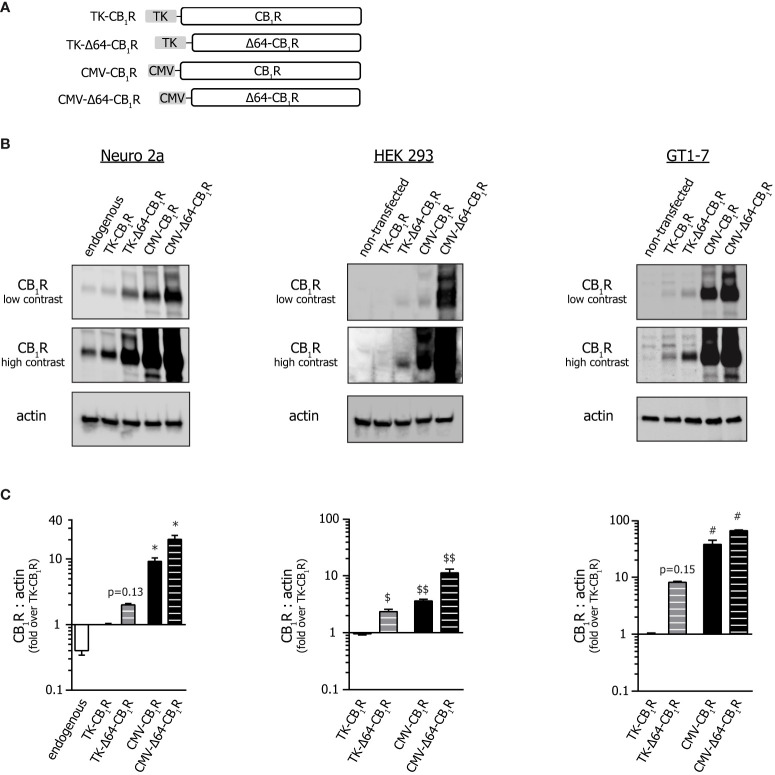
*Effect of promoter and N-terminal truncation on CB_1_R expression.* **(A)** Schematic representation of the various CB_1_R clones used throughout the study. (For GFP-tagged versions, see *Supplementary Figure 3A*.) **(B)** Western blot analysis of the expression of various CB_1_R constructs in Neuro 2a, HEK 293 and GT1-7 cells. Cells were transfected with different CB_1_R constructs or with empty plasmid (pcDNA3.1(+); ‘non-transfected’); in order to perceive the differences in expression better, both low and high contrast representations of the same 16-bit raw images are presented. **(C)** Statistical analysis of immunoblots shown on *Panel (B)*. CB_1_R to actin expression ratios were normalized to that obtained in TK-CB_1_R expressing samples. From left to right n= 9-13-14-12-11 (Neuro 2a); 14-13-11-10 (HEK 293) and 11-9-10-10 (GT1-7); *p < 0.0003, ^$^p = 0.0152, ^$$^p < 0.0001 and ^#^p < 0.0001 as compared to pertinent TK-CB_1_R group (Kruskal-Wallis ANOVA followed by Dunn’s multiple comparisons test).

In order to study the effect of expression level and the long N-terminal tail on CB_1_ receptor abundance in heterologous systems, we cloned the full-length and the N-terminally truncated (Δ64) human CB_1_R into vectors that use either the conventional, strong early-immediate CMV (cytomegalovirus) or the weak HSV (herpes simplex virus) thymidine kinase (TK) promoter for transcription initiation (**Figure 1A**). We transfected the constructs into 3 different cell types, namely into HEK 293 cells, into undifferentiated Neuro 2a neuroblasts (29) and into highly-differentiated GT1-7 neurons (30). Neuro 2a cells express functional CB_1_Rs (**Supplementary Figure 1A** and **Supplementary Figure 2A**), therefore, non-transfected Neuro 2a cells served as endogenous CB_1_R controls. As expected, TK promoter-driven CB_1_R expression was about 1-1.5 order of magnitude lower than that provided by the conventional CMV promoter (**Figures 1B, C**). In all 3 cell types, shortening the long N-terminal tail (22) by 64 amino acids also enhanced receptor expression by approx. 2 to 5-fold independently of the promoter (**Figures 1B, C**). Most importantly, however, the expression of the full-length TK-CB_1_R was comparable to that of endogenous receptors (**Figures 1B, C**) suggesting that this construct may be sufficient to provide close-to-physiological receptor levels in heterologous systems.

### Tuning Down CB_1_R Expression Is Sufficient to Prevent Its Overt Proteasomal Degradation

The instability of CB_1_R in heterologous expression systems has been primarily attributed to the receptor’s long N-terminal tail that complicates receptor positioning across the ER membrane and thus triggers rapid proteasomal degradation (22). Indeed, in harmony with previous reports (22), N-terminal ablation eliminated the sensitivity of the CMV-CB_1_R construct to the proteasome inhibitor Mg-132 (**Supplementary Figure 1C**) indicating that the full-length CMV-CB_1_R is in fact prone to ubiquitination and subsequent proteasomal degradation. Similar observations were made in HEK 293 cells as well (data not shown). However, no significant proteasomal degradation was observed with the low-expression TK-CB_1_R variants (**Supplementary Figure 1D**). Instead, these low expression receptors displayed sensitivity to chloroquine (**Supplementary Figure 1D**), an inhibitor of endolysosomal degradation, similarly to that observed for the endogenous CB_1_ receptor (**Supplementary Figure 1B**). Interestingly, despite the substantial proteasomal degradation of the CMV-CB_1_ receptor, caused most likely by abnormal folding (22), none of the constructs induced measurable ER unfolded protein response, as assessed by the phosphorylation of eukaryotic initiation factor 2 subunit α (eIF2α) (**Supplementary Figure 1E**). Thus, weak promoter-driven, low-level expression of the full-length CB_1_R precludes drastic proteasomal degradation without the need for truncation of the receptor. Additionally, weakly expressed CB_1_R variants retain endolysosomal processing resembling endogenous CB_1_Rs in this respect.

### Parallel MAPK Signalling Cascades Exhibit Different Sensitivity to CB_1_R Abundancy

**Figure 2:**
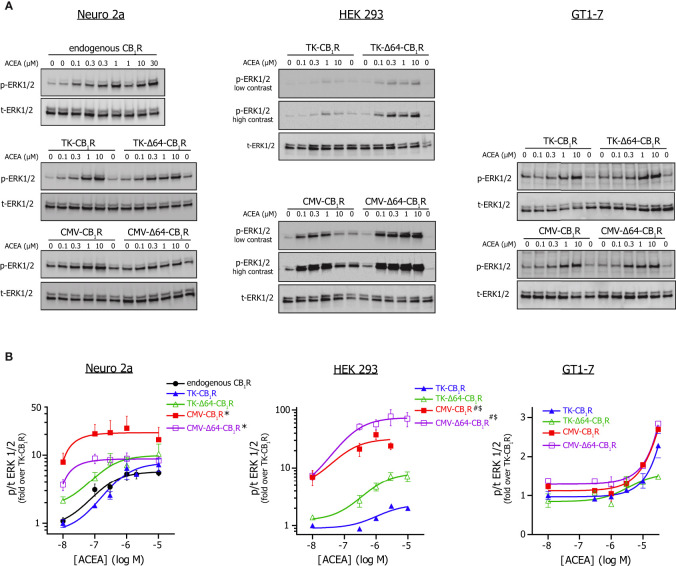
*ERK 1/2 activation by CMV or TK promoter-driven full-length and Δ64-CB_1_ receptor variants.* **(A)** Western blot analysis of ERK 1/2 phosphorylation mediated by the various CMV and TK promoter-driven or endogenous CB_1_ receptors stimulated with ACEA. Neuro 2a, HEK 293 and GT1-7 cells expressing the indicated CB_1_R variants or endogenous receptors (Neuro 2a) were stimulated with various concentrations of ACEA in at 37°C in DMEM + HEPES for 5 min. Representative western blots are shown; please note that, although presented as separate blots, p-ERK and t-ERK membranes were actually developed under identical conditions (incl. exposure times) so these images may be directly compared within the pertinent cell type. **(B)** Dose-response analysis of ACEA-evoked p-ERK 1/2 signals from western blot experiments as the one presented on *Panel (A)*. Phospho- to total ERK 1/2 values were normalized to the minimum response of the TK-CB_1_R group and the 3-parametered log[agonist] – response equation was used to fit concentration-response curves. Number of observations was 3-8/construct/ACEA concentration; *p = 0.036 for the effect of CMV promoter on basal ERK activity and p = 0.19 for the effect of CMV promoter on EC_50_ when compared to TK promoter in Neuro 2a cells; ^#^p = 0.0002 for the effect of CMV promoter on basal phosphorylation and ^$^p = 0.0288 for the effect of CMV promoter on EC_50_ value vs. TK promoter in HEK 293 cells (2-way ANOVA).

Next, we assessed whether changes in expression level translates into different signalling behaviour of the CB_1_R. To this end, we first monitored the phosphorylation of ERK1/2 (p42/44 MAPK) by western blotting. HEK 293, Neuro 2a and GT1-7 cells were stimulated with increasing concentrations of the CB_1_R specific anandamide analogue arachidonyl-2-chloroethylamide (ACEA) (31), which brings about ERK 1/2 activation in a CB_1_R dependent manner (**Supplementary Figure 2A**). In non-transfected HEK and GT1-7 cells, ACEA failed to induce ERK1/2 or p38 MAPK phosphorylation (data not shown). In Neuro 2a cells, the full length TK-CB_1_R variant produced a stimulus-response curve that practically overlapped with that produced by endogenous CB_1_ receptors in non-transfected cells (**Figures 2A, B**). On the other hand, CMV promoter-driven high expression CB_1_R variants increased the basal phosphorylation of ERK1/2 and tended to shift the dose-response curve to the left. Congruent ERK 1/2 activation data were obtained in HEK 293 cells (but not in GT1-7 neurons) (**Figure 2A, B**). In contrast, ACEA-induced activation of p38 MAPK, another downstream target of CB_1_Rs that may be recruited independently of ERK1/2 (32, 33), proved to be much less sensitive to CB_1_R abundance or N-terminal Δ64 truncation than observed for ERK1/2 phosphorylation (**Supplementary Figures 2B, C**).

**Figure 3:**
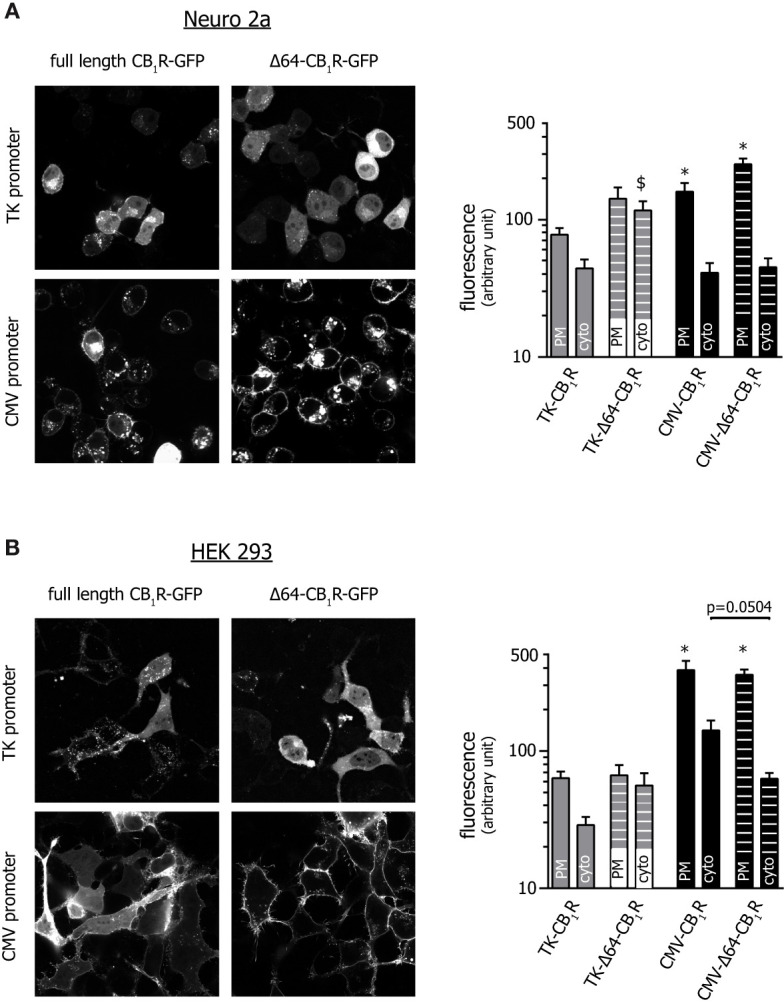
*Confocal microscopic assessment of the localization of various CB_1_R mutants.* Images of Neuro 2a **(A)** and HEK 293 **(B)** cells expressing GFP-tagged versions of the pertinent CB_1_ receptor variants were acquired with spinning disk confocal microscopy along the entire z-axis of the cell. Slices positioned at the bottom 1/3 (*i.e.* close to the cell-coverslip interface) are presented and show an area of approx. 100 x 100 µm. (Please note that due to the significant differences in expression, the brightness of images showing TK promoter-driven receptor variants was increased to a higher extent.) Bar graphs show average plasma membrane and cytosolic fluorescent intensity after background subtraction. In all groups n=16; *p < 0.05 when compared to TK-CB_1_R PM (one-way ANOVA followed by Dunn’s or Holm-Sidak’s multiple comparisons test for Neuro 2a and HEK 293, respectively), ^$^p = 0.012 *vs*. TK-CB_1_R-cyto (one-way ANOVA and Dunn’s test), for the comparison between CMV-CB_1_R-cyto and CMV-Δ64-CB_1_R-cyto one-way ANOVA and Holm-Sidak’s test were applied.

Confocal microscopy in Neuro 2a and HEK 293 cells revealed that the ability to increase basal ERK1/2 activity corresponds well to the cell surface expression of CB_1_R constructs, with CMV-CB_1_R variants having around an order of magnitude higher plasmalemmal abundance than their TK-CB_1_R counterparts (**Figures 3A, B**). Intriguingly, whereas Δ64 truncation improved plasma membrane targeting of CB_1_Rs under high-expression conditions, the same ablation did not enhance cell surface localization of weakly expressing TK-CB_1_R variants. These data together imply that CB_1_R-activated parallel MAPK signalling pathways display different sensitivity to supraphysiological CB_1_R expression, and that weak-expression recombinant CB_1_ receptor variants mimic the behaviour of the endogenous receptors more closely than conventional high-expression CB_1_R constructs.

### Low CB_1_R Expression Levels Ensure Predominant G_i/o_ Coupling

**Figure 4:**
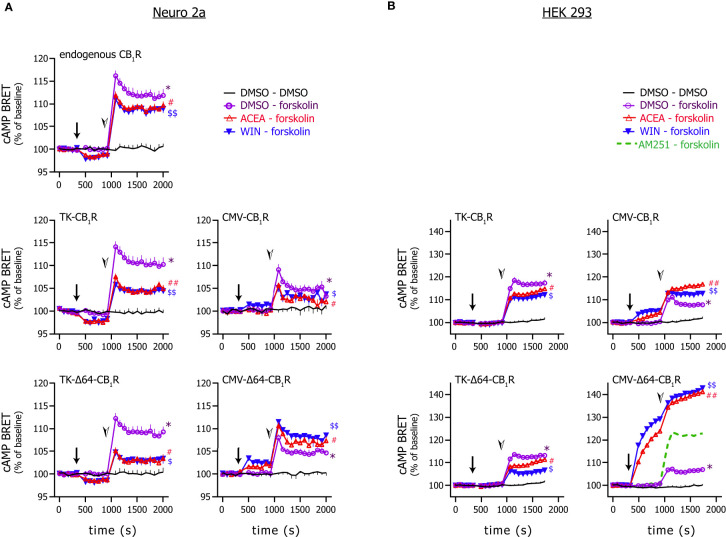
*Effect of CB_1_R stimulation on cytosolic cAMP.* Neuro 2a **(A)** and HEK 293 **(B)** cells expressing the EPAC-based intramolecular cAMP sensor together with the indicated CB_1_R variant were stimulated first with the CB_1_R agonists ACEA (20 µM) or WIN55,212-2 (1 µM) or vehicle (arrows) followed by the addition of forskolin (1 µM) or vehicle (arrowheads). BRET ratios were normalized to the average measured during control period. (In one experiment, the effect of the CB_1_R inverse agonist AM251 (2 µM) was also tested instead of CB_1_R agonist; average of 3 wells are shown.) Number of observations was min. 8 wells from 3 independent experiments. In some cases, to aid perceptibility, mean + or – S.E.M are presented only; in some graphs, symbols are larger than error bars and thus the latter are not visible. Data were analysed with 2-way ANOVA in combination with Dunnett’s multiple comparisons test. Symbols represent significances as follows: *: significant difference (p < 0.0001) *vs*. DMSO-DMSO detected after the addition of forskolin; # and $: significant difference (p < 0.0001) *vs*. DMSO-forskolin detected after the addition of forskolin; ## and $$: significant difference (p < 0.05) *vs*. DMSO-forskolin detected already after the addition of the CB_1_R agonist and before forskolin stimulation and significance increased (p < 0.0001) after the addition of forskolin.

The coupling preference of CB_1_Rs may be swayed from G_i/o_ proteins towards the G_s_ pathway by several factors (34–36) including the expression level of the receptor itself (15). Therefore, we tested the effect of ACEA and the CB_1_R full agonist WIN 55,212-2 on cytosolic cAMP in Neuro 2a neuroblasts and HEK 293 cells expressing various CB_1_R clones. In Neuro 2a cells expressing endogenous CB_1_Rs only, both ligands reduced the basal and forskolin-stimulated cAMP concentrations signifying G_i/o_ coupling. This G_i/o_ preference was clearly retained in Neuro 2a and HEK 293 cells transfected with either the TK-CB_1_R or the TK-Δ64-CB_1_R clones but was lost or shifted towards G_s_ when CB_1_R expression was driven by the CMV promoter (**Figure 4**). Thus, as seen with the other cannabinoid signalling pathways and receptor degradation, the weakly expressed full-length CB_1_Rs satisfyingly resemble the signalling behaviour of endogenous receptors.

## Discussion

Meticulous characterization of receptor-ligand interactions is vital for pharmacological research and can reduce the risk of severe side-effects and idiosyncratic drug reactions. This is well illustrated by the caveats in cannabinoid ligand development – brain-penetrant CB_1_R inverse antagonist proved to be potent anti-obesity drugs (37) but exerted serious psychiatric side effects and had to be eventually withdrawn (38, 39). Such side effects may be hidden deeply in the pharmacological properties of drugs and thus may be hard to recognize at first. Nevertheless, meticulous pharmacological profiling can overcome these pitfalls to exploit the real therapeutic potential of future pharmacons (4, 13, 40). Artificially induced expression of GPCRs in cell lines is key to deciphering almost all aspects of receptor function, and it represents a key tool for pharmacological studies. However, overexpression of a receptor may fundamentally change its biological properties such as receptor trafficking and coupling to signalling partners (15–18, 41, 42).

In the present study, we explored the role of expression level on CB_1_R function. To this end, we changed the conventional high-efficiency CMV promoter to the less effective HSV thymidine kinase promoter. The HSV thymidine kinase promoter was shown to yield significantly reduced but still detectable fluorescent protein levels, when compared to the CMV promoter (43). In our hands, TK promoter-driven weak CB_1_R expression was still sufficient to decrease cytosolic cAMP and induce detectable ERK 1/2 and p38 MAPK signalling upon CB_1_R stimulation. Furthermore, the full-length TK-CB_1_R construct was functional in 3 different cell types – HEK 293 cells, a general model of mammalian cells widely used in pharmacological research, in Neuro 2a murine neuroblasts (29) with high differentiation potential (44), and also in highly differentiated GnRH-secreting GT1-7 neurons (30).

CB_1_Rs are known to couple to several parallel downstream signal transduction pathways (5, 33, 45). Amongst these, reduction of cytosolic cAMP *via* the recruitment of the G_i/o_ heterotrimeric G-protein was the first recognized intracellular effect of cannabinoids (46) and it is still appreciated as a crucial signalling step conveying many of the therapeutic effects (or side-effects) of cannabinoids (13, 47). However, preference of CB_1_Rs may be shifted towards the G_s_ pathway by several factors (34) (35, 36), including the expression level of the receptor itself (15). Indeed, whereas endogenous receptors and low-expression recombinant CB_1_R variants displayed G_i/o_ engagement predominantly, the G protein preference of CMV-driven CB_1_Rs was switched to the non-canonical G_s_ pathway in our cAMP paradigm. This shift in cAMP signalling of cannabinoids was recognized early on (41, 48) and was further characterized in an elegant recent study (15). Finlay and co-workers used HEK 293 cell lines stably expressing the CB_1_R with either high or low efficiency to show an analogous shift in G protein preference (15). Although stable cell lines offer several advantages over transient expression, this strategy can be laborious, and it can hardly be applied for mutational analysis of the receptor, when numerous permutations are usually examined.

It is noteworthy that a change in G protein preference is not a unique feature of the CB_1_R, as coupling of several GPCRS to G proteins is sensitive to the receptor - G protein ratio (20, 21), to the available G protein pool, to net receptor density (49, 50) and possibly to the applied ligand (34, 42, 45). For instance the luteinizing hormone receptor, the V_2_ vasopressin receptor, the β_1_- and β_2_-adrenergic receptors were shown to induce inositol trisphosphate formation at high receptor counts only (51). Our present findings complement these literary data well and they together underline that receptor density, ligand properties and cell type must all be taken into account to draw reliable conclusions about physiological receptor signalling.

Phosphorylation of ERK 1/2 is a canonical effect of CB_1_R activation (33, 52), and its mechanism depends on the cell type, the agonist, on receptor internalization, β-arrestin expression pattern and on the presence of allosteric modulators (53–55). Despite this complexity, weakly overexpressed full-length and endogenous CB_1_Rs produced practically identical ERK 1/2 concentration-response curves suggesting that these receptors engage the same combination of signalling partners to initiate ERK signalling. The highly expressed CMV-CB_1_Rs, on the other hand, shifted the dose-response curves to the left, in accordance with their higher plasma membrane expression. It has to be added here that higher plasma membrane expression did not shift the concentration-response curve in all scenarios. For instance, p38 MAPK activation appeared to be less sensitive to CB_1_R density in Neuro 2a cells. This phenomenon lays out of the scope of the present study and remains to be elucidated.

CMV-driven high-expression CB_1_ receptors appear to have substantial basal activity compared to weakly expressed receptor variants. This notion is supported by i) the significant increase in basal ERK 1/2 phosphorylation ii) by the smaller forskolin-induced cAMP increase in CMV-CB_1_R expressing cells indicating basal G_s_ engagement iii) and by the formation of cytosolic protrusions in non-stimulated CMV-CB_1_R expressing Neuro 2a neuroblast (**Supplementary Figure 3**) that may signify increased basal CB_1_R activity in these cells (56, 57). Basal endocannabinoid production and CB_1_R activity is most probably inherent to most cells (58, 59) but overactive CB_1_R signalling under resting conditions may have several uncontrolled effects that need to be carefully considered when interpreting experimental results.

Similarly to signalling, the weakly expressed TK-CB_1_R also mimicked the distribution pattern of endogenous receptors. The presence of receptors in intracellular vesicle-like structures was easily notable with low-expression full length TK-CB_1_Rs, closely resembling the distribution of endogenous CB_1_Rs in Neuro 2a and primary hippocampal neurons (60). Intracellular receptors associate to both non-endolysosomal vesicles (60) and to endolysosomes as a result of internalization (58, 61). In this regard, it is noteworthy to recall that weakly expressed bradykinin type-2 receptors also display higher internalization rate than highly expressed counterparts (62).

In an elegant series of experiments, Andersson and colleagues showed that high-expression CB_1_R variants exhibit substantial proteasomal degradation that can be mitigated by truncating the long N-terminal tail (22). Our data corroborates their findings as we also observed that Δ64 modification ameliorates proteasomal degradation and improves plasmalemmal localization of CMV-CB_1_R variants. We further extended this paradigm by showing that tuning down the expression level *alone* is sufficient to redirect CB_1_ receptors from proteasomal degradation pathways towards endolysosomal processing, which is characteristic of endogenous receptors (33). Intriguingly, as opposed to high-expression variants, Δ64 deletion in low expression TK-CB_1_R variants increased the diffuse cytoplasmic fluorescence, implying that the long N-terminal tail may be a limiting factor of normal ER translocation under high-expression conditions only. Altogether, the weakly expressed full-length CB_1_R mimics the intracellular distribution as well as the receptor degradation properties of the endogenous receptor reasonably well.

In conclusion, our data demonstrate that using vectors with low efficiency promoters for the heterologous expression of CB_1_Rs is a favourable option for studying cannabinoid ligands and CB_1_R function. Low-level CB_1_R expression provides receptor distribution, G_i/o_, ERK 1/2 and p38 MAPK signalling that are comparable to that observed with endogenous receptors, and precludes non-canonical signalling and overt proteasomal degradation. Whether these benefits also extend to other CB_1_R-mediated signalling events, such as ceramide production, G_q_ recruitment or protein-tyrosine kinase activation, needs to be elucidated by future studies.

## Data Availability Statement

The raw data supporting the conclusions of this article will be made available by the authors, without undue reservation.

## Author Contributions

VH, AT, and GS conceived and designed the study. VH and GS performed the majority of experiments and analysed most of the data. ES-K, AT, and GS planned, carried out and analysed cAMP measurements. ÉW assisted with molecular biological work. AR and EH cultured the cells. AR performed transfection, western blot, and analytical work. BE performed confocal microscopy. LH contributed to data analysis, manuscript amendment, discussion, and proofreading. GS, AT, and VH wrote the MS. All authors read the manuscript and agreed with the final version.

## Funding

This work was supported by the following grants: National Research, Development and Innovation Office grants (NKFI-6/FK_124038) to GS and by the Scientific and Innovation Fund (26303/AOELT/2019) of the Semmelweis University, Budapest, Hungary, to GS. The laboratories were also funded by the Hungarian National Research, Development and Innovation Fund (grant numbers: NVKP_16-1-2016-0039 and NKFI K116954) to LH. BE was supported by a “Lendület” grant from the Hungarian Academy of Sciences (LP2018-13/2018) and funding form EU’s Horizon 2020 research and innovation program (grant agreement No. 739593). The Department of Physiology also received funds from Higher Education Institutional Excellence Programme (FIKP) of the Ministry of Human Capacities in Hungary, within the framework of the Molecular Biology thematic programme of the Semmelweis University.

## Conflict of Interest

The authors declare that the research was conducted in the absence of any commercial or financial relationships that could be construed as a potential conflict of interest.

## Publisher’s Note

All claims expressed in this article are solely those of the authors and do not necessarily represent those of their affiliated organizations, or those of the publisher, the editors and the reviewers. Any product that may be evaluated in this article, or claim that may be made by its manufacturer, is not guaranteed or endorsed by the publisher.

## Supplementary Material

Supplementary Figure 1
**(A)** Neuro 2a cells express CB_1_R endogenously as assessed by siRNA-mediated knock-down. Cells were transfected with siRNA directed against murine cnr1 or with a minimally-altered non-silencing double-stranded RNA (control dsRNA) and with various CB_1_R mutants or empty plasmid as indicated. Actin immunoreactivity was used as loading control. Representative for 3 independent experiments (p < 0.05 for the effect of siRNA on CB_1_R expression; ANOVA and Holm-Sidak’s test). **(B)** Endogenous CB_1_Rs in Neuro 2a cells are primarily processed/degraded in the endolysosome system. Neuro 2a cells were treated with the proteasome inhibitor MG-132, the lysosomal inhibitor chloroquine (200 µM) or with DMSO (vehicle) for 8 h, as indicated, and CB_1_R protein expression was evaluated with immunoblotting. Bar graph represents the ratio of CB_1_R band density between ~50-100 kDa to that below 40 kDa. n = 4 in all groups; *p < 0.05 when compared to vehicle (Kruskal-Wallis ANOVA followed by Dunn’s multiple comparisons test). **(C)** The high-expression CMV-CB_1_R variant exhibits high turnover proteasomal degradation while TK promoter-driven receptors are processed in the endolysosomal system. GT1-7 neurons transfected with the CMV (left and middle blots) or TK (right blots) promoter-driven full-length or Δ64-CB_1_ receptor variants were exposed to the lysosomal inhibitor chloroquine (200 µM), the proteasome inhibitor Mg-132 (200 nM), the calpain and cathepsin B inhibitor MDL-28170 (25 µM), or the trypsin-like/cysteine protease inhibitors E64 (20 µM) and leupeptin (10 mg/mL) for 8 h and CB_1_R expression pattern was analysed by western blotting. For statistical analysis, see *Panel D*. **(D)** Statistical analysis of blots shown on *Panel C*; ratio of CB_1_R band density between ~50-100 kDa to that measured below 40 kDa were evaluated. Number of observations was 4 in all groups except for CMV-Δ64-CB_1_R n=3; *p < 0.05 *vs*. vehicle control (one-way parametric or Kruskal-Wallis ANOVA with Holm-Sidak’s or Dunn’s post-hoc tests, respectively). **(E)** None of the recombinant CB_1_R variants induce significant ER stress response. Neuro 2a, HEK 293 and GT1-7 cells transfected with various CB_1_R constructs or empty plasmid (non-transfected) were analysed for ER stress by monitoring eIF2α phosphorylation with immunoblotting. As positive control, non-transfected Neuro 2a cells were exposed to 200 nM thapsigargin, an inhibitor of SERCA Ca^2+^ pumps, for 4 h in serum-free DMEM. Number of observations from left to right, Neuro 2a n = 4-4-9-9-9-9, HEK 293 n=3 in all groups, GT1-7 n = 4-5-5-5-5; *p < 0.0001 *vs*. non-transfected DMSO treated group (ANOVA and Holm-Sidak’s test).

Supplementary Figure 2
**(A)** ACEA-evoked ERK 1/2 activation requires the presence of CB_1_Rs. Neuro 2a cells were transfected with non-silencing dsRNA or with siRNA directed against endogenous CB_1_Rs or exposed to transfection reagent only (‘no dsRNA’) and were stimulated with the anandamide analogue ACEA for 3 min. Representative for 2 independent experiments; p = 0.035 for the effect of siRNA on phospho-ERK 1/2 response (Mann-Whitney test; n = 5 and 4 for dsRNA treated ACEA exposed and siRNA treated ACEA exposed). **(B)** Western blot analysis of p38 MAPK phosphorylation evoked by ACEA *via* the different CMV and TK promoter-driven and endogenous CB_1_ receptors stimulated with ACEA. Neuro 2a, HEK and GT1-7 cells expressing the indicated CB_1_R variants or endogenous receptors (Neuro 2a) were stimulated with various doses of ACEA in at 37°C in DMEM + HEPES for 5 min. Representative western blots are shown. Please note that, although presented as separate blots, p-ERK and t-ERK membranes were actually developed under identical conditions (incl. exposure times) so these images may be directly compared within the pertinent cell type. **(C)** Dose-response curves for p38 MAPK activation calculated as described for Fig.2.B. Number of observations was min 3/construct/[ACEA].

Supplementary Figure 3
**(A)** Structure of GFP-tagged CB_1_R constructs used in microscopic experiments. **(B)** CMV promoter-driven heterologous expression of CB_1_Rs triggers extensive filopodium formation in Neuro 2a and HEK 293 cells as compared to TK-CB_1_R constructs. Images along the entire z-axis of cells were obtained as described for *Figure 3*. To give a 2-dimensional representation of the entire cell, z-stacks were compressed into a single image using the ZProjection-maximal intensity algorithm of Image J (Fiji version). (Please note again that the brightness of images showing TK-CB_1_R variants was increased stronger than of CMV images for representative purposes).
