# Supplementary figures and images for: Optimization of the Heterologous Expression of the Cannabinoid Type-1 (CB1) Receptor
Source: Front Endocrinol (Lausanne). 2021 Oct 20;12:740913. doi: 10.3389/fendo.2021.740913 (PMC8564136; doi:10.3389/fendo.2021.740913)

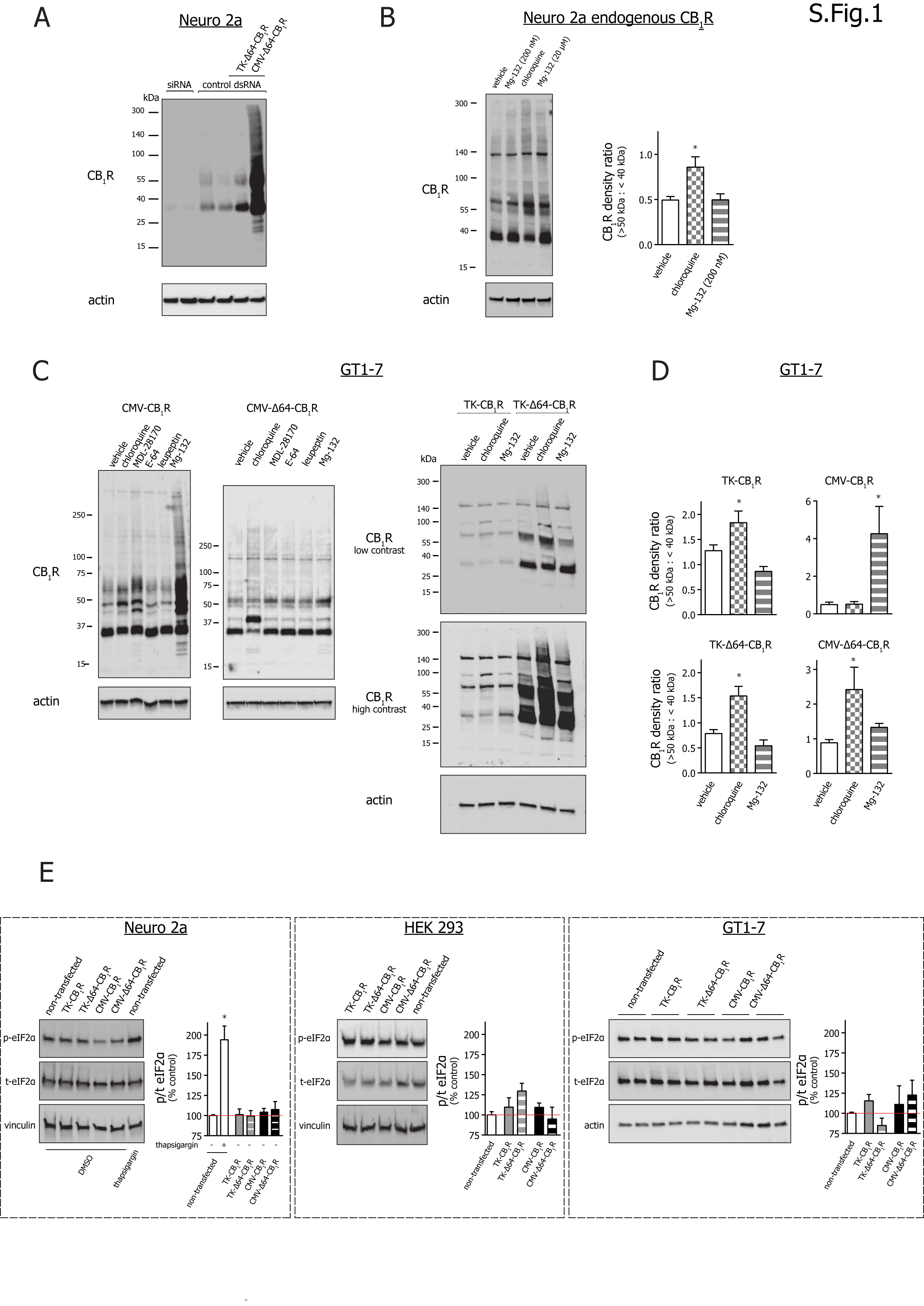

Supplement: Supplementary Figure 1 — (A) Neuro 2a cells express CB1R endogenously as assessed by siRNA-mediated knock-down. Cells were transfected with siRNA directed against murine cnr1 or with a minimally-altered non-silencing double-stranded RNA (control dsRNA) and with various CB1R mutants or empty plasmid as indicated. Actin immunoreactivity was used as loading control. Representative for 3 independent experiments (p < 0.05 for the effect of siRNA on CB1R expression; ANOVA and Holm-Sidak’s test). (B) Endogenous CB1Rs in Neuro 2a cells are primarily processed/degraded in the endolysosome system. Neuro 2a cells were treated with the proteasome inhibitor MG-132, the lysosomal inhibitor chloroquine (200 µM) or with DMSO (vehicle) for 8 h, as indicated, and CB1R protein expression was evaluated with immunoblotting. Bar graph represents the ratio of CB1R band density between ~50-100 kDa to that below 40 kDa. n = 4 in all groups; *p < 0.05 when compared to vehicle (Kruskal-Wallis ANOVA followed by Dunn’s multiple comparisons test). (C) The high-expression CMV-CB1R variant exhibits high turnover proteasomal degradation while TK promoter-driven receptors are processed in the endolysosomal system. GT1-7 neurons transfected with the CMV (left and middle blots) or TK (right blots) promoter-driven full-length or Δ64-CB1 receptor variants were exposed to the lysosomal inhibitor chloroquine (200 µM), the proteasome inhibitor Mg-132 (200 nM), the calpain and cathepsin B inhibitor MDL-28170 (25 µM), or the trypsin-like/cysteine protease inhibitors E64 (20 µM) and leupeptin (10 mg/mL) for 8 h and CB1R expression pattern was analysed by western blotting. For statistical analysis, see Panel D. (D) Statistical analysis of blots shown on Panel C; ratio of CB1R band density between ~50-100 kDa to that measured below 40 kDa were evaluated. Number of observations was 4 in all groups except for CMV-Δ64-CB1R n=3; *p < 0.05 vs. vehicle control (one-way parametric or Kruskal-Wallis ANOVA with Holm-Sidak’s or Dunn’s post-hoc t [file Image_1.tif]

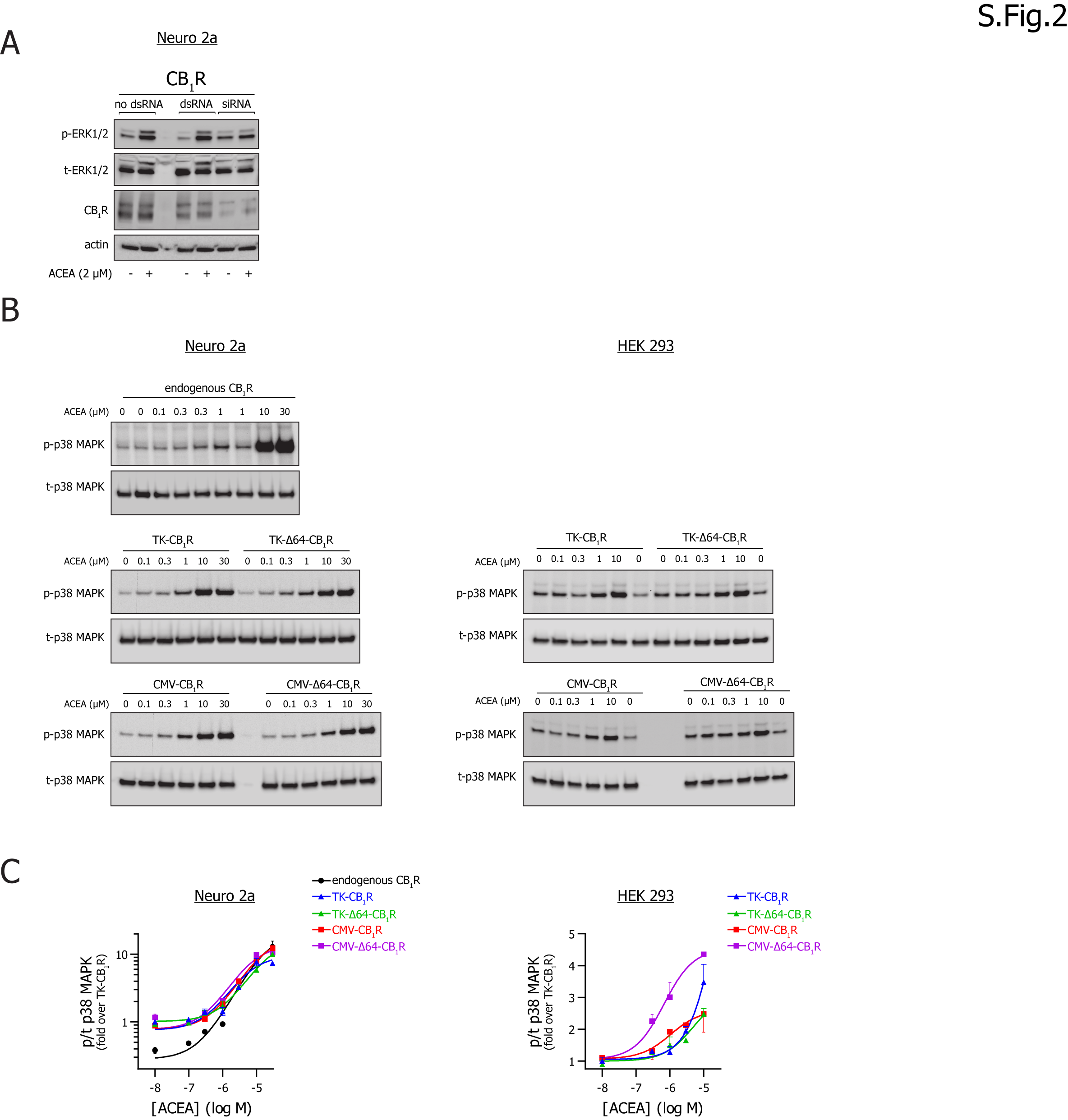

Supplement: Supplementary Figure 2 — (A) ACEA-evoked ERK 1/2 activation requires the presence of CB1Rs. Neuro 2a cells were transfected with non-silencing dsRNA or with siRNA directed against endogenous CB1Rs or exposed to transfection reagent only (‘no dsRNA’) and were stimulated with the anandamide analogue ACEA for 3 min. Representative for 2 independent experiments; p = 0.035 for the effect of siRNA on phospho-ERK 1/2 response (Mann-Whitney test; n = 5 and 4 for dsRNA treated ACEA exposed and siRNA treated ACEA exposed). (B) Western blot analysis of p38 MAPK phosphorylation evoked by ACEA via the different CMV and TK promoter-driven and endogenous CB1 receptors stimulated with ACEA. Neuro 2a, HEK and GT1-7 cells expressing the indicated CB1R variants or endogenous receptors (Neuro 2a) were stimulated with various doses of ACEA in at 37°C in DMEM + HEPES for 5 min. Representative western blots are shown. Please note that, although presented as separate blots, p-ERK and t-ERK membranes were actually developed under identical conditions (incl. exposure times) so these images may be directly compared within the pertinent cell type. (C) Dose-response curves for p38 MAPK activation calculated as described for Fig.2.B. Number of observations was min 3/construct/[ACEA]. [file Image_2.tif]

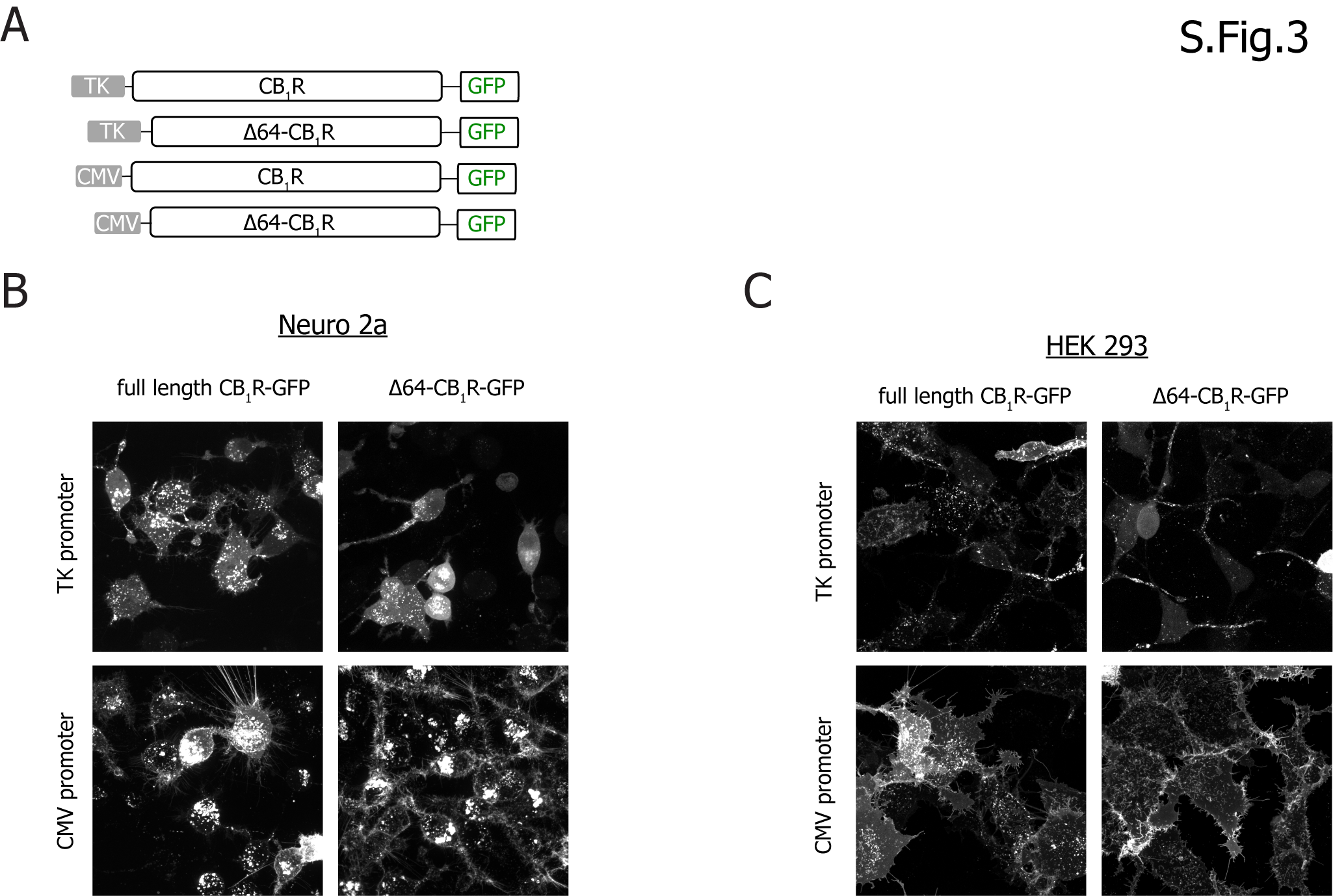

Supplement: Supplementary Figure 3 — (A) Structure of GFP-tagged CB1R constructs used in microscopic experiments. (B) CMV promoter-driven heterologous expression of CB1Rs triggers extensive filopodium formation in Neuro 2a and HEK 293 cells as compared to TK-CB1R constructs. Images along the entire z-axis of cells were obtained as described for Figure 3. To give a 2-dimensional representation of the entire cell, z-stacks were compressed into a single image using the ZProjection-maximal intensity algorithm of Image J (Fiji version). (Please note again that the brightness of images showing TK-CB1R variants was increased stronger than of CMV images for representative purposes). [file Image_3.tif]
